# Beyond the “Third Wave of Positive Psychology”: Challenges and Opportunities for Future Research

**DOI:** 10.3389/fpsyg.2021.795067

**Published:** 2022-01-14

**Authors:** Marié P. Wissing

**Affiliations:** Africa Unit for Transdisciplinary Health Research, Faculty of Health Sciences, North-West University, Potchefstroom, South Africa

**Keywords:** positive psychology, beyond “the third wave”, post-disciplinarity, complex phenomena, metatheoretical assumptions, theory, methodological reform, harmony

## Abstract

The positive psychology (PP) landscape is changing, and its initial identity is being challenged. Moving beyond the “third wave of PP,” two roads for future research and practice in well-being studies are discerned: The first is the state of the art PP trajectory that will (for the near future) continue as a scientific (sub)discipline in/next to psychology (because of its popular brand name). The second trajectory (main focus of this manuscript) links to pointers described as part of the so-called third wave of PP, which will be argued as actually being the beginning of a new domain of inter- or transdisciplinary well-being studies in its own right. It has a broader scope than the state of the art in PP, but is more delineated than in planetary well-being studies. It is in particular suitable to understand the complex nature of bio-psycho-social-ecological well-being, and to promote health and wellness in times of enormous challenges and changes. A unique cohering focus for this post-disciplinary well-being research domain is proposed. In both trajectories, future research will have to increase cognizance of metatheoretical assumptions, develop more encompassing theories to bridge the conceptual fragmentation in the field, and implement methodological reforms, while keeping context and the interwovenness of the various levels of the scientific text in mind. Opportunities are indicated to contribute to the discourse on the identity and development of scientific knowledge in mainstream positive psychology and the evolving post-disciplinary domain of well-being studies.

## Introduction

The positive psychology (PP) landscape is broadening and changing in terms of assumptions, methods, and empirical focus. In this perspective manuscript, I will argue that the so-called “third wave of positive psychology” was actually the beginning of a new multi-, inter- or transdisciplinary domain of study focusing on well-being as multimodal with a focus on humans, but also beyond the individual and human social systems, and in particular suited to understand and promote health and well-being in complex situatedness. I use the construct “well-being” in this manuscript as an umbrella term for various facets of positive functioning and being denoting something “good” taking situation and context into account. It will be indicated that there are, for purposes of this manuscript (at least) two main roads in well-being studies ahead, with some similar but also widely unique challenges and opportunities. The first is the accumulation of knowledge in the state-of-the-art PP as a scientific discipline in/next to psychology. The second is the development of a post-disciplinary trajectory for which the signs were noticed in the so-called third wave of PP (“post-disciplinary” refers in this manuscript to studies in which more than one discipline is involved, and includes multi-, inter- and transdisciplinary approaches; the latter three being increasingly integrative in focus and cooperation among contributors). It has yet to be established further to what extent this suggested post-disciplinary research domain dovetail, differ or overlap in focus and goals with some other trends studying well-being in broader contexts (e.g., the planetary health perspective, the science of effective well-doing, or the emerging science of virtues). It will be argued that future research in both trajectories will need to take the interwovenness of all the levels of the scientific text into account.

The changing face of PP can also be seen as a question about the identity and goal of PP as a scientific field, and how it will be changing further in research and practice. In the early days of PP, there was some speculation whether it reflects a paradigm shift in psychology or whether it is only a new movement in the field of psychology. With the flourishing of empirical research in PP, this more metatheoretical scientific question was not revisited until recently, while there was a rapid growth in empirical studies. Nowadays, it is still unclear whether PP is a separate domain of scientific study or whether it should be seen as a sub-discipline in (the disunity of) psychology or whether it should be integrated into all existing sub-divisions in psychology. All of these perspectives can be found in the PP literature. Findings on facets and dynamics of well-being are at present to be found within many branches of psychology itself, for example, in clinical, counseling, educational and organizational psychology, developmental psychology, community psychology, social psychology, personality psychology, and health psychology, and in future PP may even be integrated into psychology. Nevertheless, PP is not explicitly owned up in mainstream psychology (there is even tension between proponents of psychology vs. positive psychology about some issues such as practice and professionalization). At the moment there is to some extent, consensus that PP has over time established itself as a separate scientific (sub)discipline. The focus in PP was thus far on the psychological well-being of humans and human systems, and how context can influence that. However, not enough attention has been paid to how humans also contribute or detract from the well-being of non-human and ecological systems themselves, and how the coherence of all of these can be optimized. New research streams are also involved in well-being studies, with a broader goal and focus, and these may not fit well under the flag of PP.

It is time for a thoughtful reflection on the development and identity of PP and that of other well-being studies with emerging identities and scope to be further clarified as the post-disciplinary well-being trajectory proposed in this manuscript. Such reflection will require awareness and explication of metatheoretical assumptions about reality and what knowledge production entails, which is driving both focus and methods (Alexandrova, 2017; Hill and Hall, 2018). Today’s complex problems require a holistic view of the many facets and layers of human and contextual well-being with many disciplines involved and a broader scope than the individual person. It is a question of whether the post-disciplinary scientific field focusing on well-being and positive health as described in the so-called third wave of PP, will branch out of PP into a new dominantly inter-, multi- and transdisciplinary domain with a new focus as suggested in this manuscript, or whether it will dissolve back into PP which is more about the proliferation of detailed and in-depth research on (fragments of) psychosocial well-being, or if both trajectories will develop side-by-side. Apart from the mono- vs. multidisciplinary nature of these scientific endeavors, it is also a question whether the goal will be well-being of the individual and human groups, or whether the well-being of the wider contextual systems themselves as resources for all life on earth is part of the envisioned future, and to what extent it will be.

## Thought Developments in Positive Psychology as Science

### Science Development

There are various perspectives on the development of science of which only some will be highlighted here with reference to the development of PP and other well-being studies. Positive psychology as a scientific discipline developed similarly to many other scientific disciplines in the process of knowledge accumulation through continuous differentiation and integration of information as conceptualized and described by Staats (1999) for psychology as a discipline. However, apart from the rapid accumulation of knowledge in PP, some shifts took place on metatheoretical, theoretical and empirical levels akin to conceptualizations by Kuhn, a philosopher of science. Kuhn (1970, 1977) proposed the idea of a “disciplinary matrix” which refers to the normative and shared beliefs among most scientists in a discipline. Such beliefs include ontological and epistemological assumptions, values, and a typical focus and vocabulary in research. Kuhn distinguished between the so-called normal and revolutionary phases in scientific development, with major shifts in the latter phase. In the “normal phase” of science there is an accumulation and growth of knowledge. However, in the development of a discipline, researchers sometimes come across problems that they struggle to solve. After fruitless efforts, a new phase, the so-called “revolutionary phase” emerges in which major changes occur. New assumptions and perspectives are embraced, new foci and methods found, and new theories developed. This is what Kuhn called a “paradigm shift.” Such shifts tend to take place on all levels of the “scientific text” as distinguished by Madsen (1988): on a philosophical (ontological and epistemological) level, a theoretical level, and on the empirical level. Apart from changes in components of the disciplinary matrix, the direction of research may also change. Although the paradigm shifts conceptualized by Kuhn (1970, 1977) can to some extent be noticed in the natural sciences, they are not as sharply delineated in the human and social sciences. Nevertheless, changes and shifts occur in the social and human sciences, but they typically develop more gradually, and previous ideas may co-exist with new perspectives (Madsen, 1988), as is the case in the various phases distinguished in the development of PP and the understanding of well-being.

Van den Besselaar (2012/2018) touched upon the issue of disciplinary and interdisciplinary research identities in his perspective on forms of change in knowledge systems. He contends that the cognitive identity of a discipline precedes the theories and methods used in that discipline, and that disciplines can be defined in terms of the questions that guide the research, instead of only the subject area. From this viewpoint, new questions can facilitate various patterns of knowledge development and change that can include splitting within the field (specialization – as what the case for PP might have been), growth in the original discipline, or decline in interest in the field, merging with other disciplines (integration), or the birth of a new field of study (as the case of the proposed new domain of post-disciplinary well-being studies may be), and more. He contends that interdisciplinarity is one of the ways in which research fields develop, but sees it as a temporary phase in the dynamics of knowledge development. Such interdisciplinary studies emerge at the boundary of an existing field in the context of multidisciplinary research activities. From here it can develop into a mature science on its own (a “new” discipline), or remain for some time as multidisciplinary or interdisciplinary, or after a while disappear. Such a multidisciplinary or interdisciplinary field of study may also grow into a *transdisciplinary* research domain which can eventually be seen as a mature field of study. However, the proposed post-disciplinary trajectory of well-being studies discerned in this manuscript is now seen as a new field of study being born, which needs further growth before reaching the status of a mature scientific domain.

### Phases of Development in Positive Psychology

It is noteworthy that conceptualizations of (facets of) well-being can be found in psychology and physical health related literature and textbooks long before the formal recognition of PP as a field of study, with many of these later on integrated into PP. Examples of such ideas are those by Jahoda (mental health), Maslow (self-actualization), Rogers (the optimally functioning person), Allport (maturity), and Frankl (the will to meaning). Antonovsky (1987) explicitly defined health not only in terms of the absence of disease, but also in terms of the presence of positive (salutogenic) characteristics, and resilience in adverse situations was already described by Rutter (1987). Other important forerunners were Diener (1984) focusing on subjective well-being and satisfaction with life, Deci and Ryan (1985) formulating the self-determination theory, and Ryff (1989) postulating her model on psychological well-being. There are many more paving the way for what is now known as positive psychology, exploring what is best in people and how to promote it. A full review is beyond the scope of this manuscript. In this section I will only focus on a brief summary of developments in PP since its “formal” recognition in 1998, indicating some major shifts discernible in empirical focus, methods and metatheoretical assumptions relevant to the main argument of this manuscript, namely that the so-called third wave of PP may actually be a new domain of well-being research.

Various phases or waves had been noted in the exponential development of PP as science since its formal introduction by Seligman in his presidential address to the American Psychological Association in 1998, and the follow-up publication by Seligman and Csikszentmihalyi (2000) in the January issue of the *American Psychologist* devoted to the idea of a focus on the positives in human functioning. From the beginning (indicated retrospectively as the first wave of PP), this field attracted the attention of many researchers, practitioners and consumers around the world and a flourishing research domain developed, and is still continuing to do so. Wong (2011), Lomas and Ivtzan (2016) described what became known as the second wave of positive psychology taking both the positives and negatives of life into consideration. Wissing (2018, 2020), Wissing et al. (2018, 2021) highlighted the rise of a third wave of PP as manifested in multi and interdisciplinary approaches and shifts with regard to metatheoretical assumptions (worldviews), empirical foci and methods alongside and overlapping with the continuing so-called first and second waves. Similar observations and conceptualizations were also put forward by Lomas et al. (2021), explicitly focusing on broadening the scope and methodology of PP and elaborating on the idea of “waves.” The distinguished phases or waves of scientific development can summarily be described as follows for substantiation of arguments and relevant references, see Wissing et al. (2018, 2021), Wissing (2020), Lomas et al. (2021):

In the **first phase of PP** (emerging more or less 1998/2000 – 2010) there was a drive for research on positive aspects of human functioning in contrast to the past state of the art in psychology that mainly focused on the negatives. Researchers differentiated and explored the nature and dynamics of many components of well-being (such as satisfaction with life, positive emotions, character strengths, meaning, mindfulness, and many more), and many measures were developed for various constructs. Indices of life satisfaction soon became the golden index for the global construct of well-being. Similarities and differences between constructs were explored and higher-order integrations were made (for example, in terms of the hedonic and eudaimonic perspectives - cf. Keyes, 2002; Deci and Ryan, 2008), theories were developed (e.g., Fredrickson’s broaden and build model, 1998, 2001), and theories on well-being facets, preceding the formalization of PP, were integrated for example those by Deci and Ryan (1985), Ryff (1989, 1995), and Csikszentmihalyi (1997). Apart from empirical and theoretical work, research on the effects of positive interventions was conducted. The field of PP expanded rapidly (cf. Hart and Sasso, 2011) and Rusk and Waters (2013) showed its significant growth not only in size, but also in reach and impact. The above mentioned lines of first wave PP research are still branching out and form the bulk of present day PP research, but with some individual researchers also shifting in perspectives. However, in the early phase of PP, little attention was given to the multimodal nature of well-being. Before and during this period similar streams of well-being research also took place with researchers working in silos and with no interaction or recognition of each other’s outputs and perspectives (for example, apart from mainstream PP, similar research took place in the quality of life movement, the asset-based approach, and the positive youth development framework steered by the Search Institute, and in humanistic psychology). From the beginning there was a stand that PP should adhere to the highest principles of science: Seligman and Csikszentmihalyi (2000) stressed that “real scientific methods” should be used as in the natural sciences to unearth so-called objective truths (and avoid being seen as trivial), reflecting an (implicitly accepted) positivist philosophical approach. Mainly quantitative methodologies were used, hypotheses tested, and generalizations made, but without considering the context. Naturalist and individualist perspectives informed research. There was to a great extent ignorance concerning cultural contexts and the associated values and ethical imperatives, assuming that findings from individualist contexts will also be valid in non-individualist contexts, and that researchers are value-free. This is typical of what Henrich et al. (2010) described as WEIRD research (i.e., from Western, Educated, Industrialized, Rich and Democratic countries). In this process, no attention was paid to worldview (ontological and epistemological) assumptions which attracted critique (for example by Held, 2002, 2004; Lazarus, 2003; Christopher and Hickinbottom, 2008).

The above critiques were gradually accepted, and a **second wave of PP** emerged (more or less 2010-2015 while research typical of the first wave still continue). The second wave of PP is mainly characterized by the recognition that the positive and negative facets of life are intertwined, that well-being facets may simultaneously have positive and negative connotations, and that the interpretation of something as positive or negative depends on the cultural context and specific situatedness. This integrative view was initially proposed by Wong (2011), indicating it as PP 2.0, and then also elaborated on by Lomas and Ivtzan (2016) as “the second wave,” with many other authors indicating similar understandings. The meaning and implications of the second wave of PP was illustrated in the handbook by Ivtzan et al. (2016) in a nuanced approach illuminating the dance of the positives and negatives in life and how the “darker” experiences can also sometimes lead to positive growth. The relevance of situatedness was strongly illustrated by McNulty and Fincham (2012) amongst others, and the importance of cultural context for understanding the nature of well-being became increasingly recognized by many (e.g., Uchida and Ogihara, 2012). In this phase, more holistic well-being theories were proposed (e.g., Lomas et al., 2015). Other disciplines, apart from PP/psychology, started to explore facets of positive functioning and wellness. Recognition of the multidimensional nature of well-being started, and qualitative and mixed methods approaches were embraced. In the second wave, an awareness of the role of worldviews begin to emerge, and there was a recognition that researchers are not value-free. Constructivist and interpretivist approaches came to the foreground, while mainstream PP of the first wave was continuing.

Another shift in foci, methods and metatheoretical assumptions occurred (more or less from 2015 and onward), while research and practice in line with the first and second waves of PP were still expanding. This shift was noted and named “**the third wave” or PP 3.0** by Wissing (2018), Wissing et al. (2018), and around the same time also independently identified and developed by Lomas et al. (2021). These author groups noted many similar characteristics of the third wave (e.g., the multidisciplinary nature thereof, and changes with regard to scope/focus and methodology), but also accentuating different facets (e.g., denotations of what is meant by scope/focus, and the degree to which metatheoretical perspectives are highlighted). The so-called third wave of PP has been recognized by others focusing on well-being in various contexts (e.g., Mayer and Vanderheiden, 2020). In general, the most outstanding features of the third wave as described were a recognition of the multimodalness of well-being in an ever-changing environment and the need to move to post-disciplinarity in research, practice and education because of widespread global challenges. Lomas et al. (2021, p. 4) described the emerging third wave of PP as “a general movement of broadening “beyond the individual,” moving toward greater complexity. This includes complexity in terms of the: focus of enquiry (becoming more interested in super-individual processes and phenomena); disciplines (becoming more interdisciplinary); culture (becoming more multicultural and global); and methodologies (embracing other ways of knowing).” They highlighted the different manifestations of what is indicated as “broadening” in terms of examples of the expansion in scope and methodologies, indicating that these can also overlap. Expansion of scope included “approaches that are contextual, system informed, cultural and linguistic, and ethical,” while expansion of methodology included “greater use of qualitative methods, implicit methods, and computational science” (p. 4).

The characteristics of the third wave were described by Wissing (2018, 2020), Wissing et al. (2018, 2021) in terms of focus, methods and worldviews (ontological, epistemological and axiological) also accentuating multi- and transdisciplinarity, interconnectedness, contextualization and complexity. They highlighted the characteristics of the third wave as will be described in the following section: In the third wave, “context” was conceptualized more broadly, and included apart from the cultural context, also physical situatedness, socio-political, economic and other contexts. Wissing et al. (2018, 2021) further noted that the third wave manifested a deeper exploration and understanding of the dynamics of positives and negatives in well-being experiences, for example, in cultural contexts (e.g., Miyamoto et al., 2017), in the context of illness and suffering (e.g., Delle Fave et al., 2017; Fowers et al., 2017), as well as in interventions aiming to reduce negative symptoms while also enhancing well-being (e.g., Geerling et al., 2020; Hendriks et al., 2020). On an individual level the link between biological and psychological processes in well-being was explored from an interconnectedness approach (e.g., Delle Fave, 2018), and on a social level issues such as justice, values, ethics of care and power relationships in understanding and promoting of well-being came into focus (e.g., Di Martino et al., 2017) – highlighting the importance of wider social and political dynamics in health and well-being. On an ecological level, the quality of connectedness to the natural environment for sustainable well-being was accentuated (e.g., Helne and Hirvilammi, 2015), and the importance of spiritual connectedness was indicated (e.g., Villani et al., 2019). Many authors argued for taking into account the individual, group, society, eco-system, and spiritual levels of well-being, as well as the connections among levels (e.g., Galderisi et al., 2017; Harrell, 2018; Warren and Donaldson, 2018). These developments underscored the necessity of multi-, inter- and transdisciplinary research and practice and the use of a systems approach when engaging with complex well-being issues. However, applying this approach in complex contexts such as inequalities, migration of people, climate change, pandemics, and even in ordinary life need much further theory-to-praxis.

In the third wave epistemological assumptions made space for a variety of approaches. Mixed-methods approaches and action research became popular alongside traditional quantitative and qualitative methods, and renewed attention was paid to laypeople’s perspectives. On a metatheoretical level, various worldviews co-existed, while relational ontological assumptions were widely assumed. Ethical aspects and values were considered crucial in understanding well-being (ethics is always about the quality of relationships and responsibilities). The implications of Aristotelian virtue ethics for theory and practice were foregrounded in conceptualizations by many well-being researchers (e.g., Fowers, 2016; Proctor, 2019; Berg, 2020; Intelisano et al., 2020). A cosmodern metatheoretical perspective (e.g., Nicolescu, 2015) assuming the interconnectedness of all things also promoted integration and cooperation among disciplines, and forward a transdisciplinary approach.

The shift to post-disciplinarity in the third phase of PP is a major one, and can be seen as more pronounced than the shift from the first to the second wave of PP. It potentially signifies the development of a new direction in well-being studies. This contention will be explicated and unpacked in the following sections.

## Challenges and Opportunities Beyond the “Third Wave of Positive Psychology”

### A Third and Next Wave of Positive Psychology or a Butterfly?

A crucial question is whether the third wave of PP or that what will develop further from it, is or will be, really only (positive) psychology in its scientific/disciplinary identity. Lomas (2021) considered the possibility of a “fourth wave” of PP that will include the well-being of non-humans, but it is a question whether this is still psychology or rather a broader domain of well-being studies in development. Many streams of research and disciplines are converging in a focus on well-being issues, for example, sociology, anthropology, economy, psychology, philosophy, religion-studies, biodiversity studies, sustainability studies and biological sciences (cf. Naeem et al., 2016; Alexandrova, 2017; Delle Fave et al., 2017; Rojas, 2018; Intelisano et al., 2020; Helne, 2021; Mead et al., 2021b). Developments toward transdisciplinarity had already been ongoing for some time apart from what is described in the “third wave of PP” (Gidley, 2010; Dielman, 2015; Nicolescu, 2015; Finkenthal, 2016), and what is happening in this phase is actually a similar pattern of multidisciplinary knowledge development. Therefore, the radical changes in the shift to the “third wave of PP” might have signaled the emergence of a new scientific domain with a broader scope. This shift reflects amongst others, the recognition of the multimodalness of wellness (psychological, social, spiritual, and biological), but initially with mainly a focus on the individual and how wider systems influence the person as such. However, given the increasingly urgent demands from complex human-social-ecological problems such as consequences of climate change (floods, droughts, famine, and more), displacement of peoples (e.g., because of ongoing wars or internal country politics or economic situations), pandemics (such as COVID-19) and inequities (e.g., in resources and opportunities) with all its human well-being and health, social, socio-political, economic, and natural ecological and biosphere concomitants, there is a call for attention also to the well-being of non-humans and ecological systems for the survival of all. A single discipline cannot meet these demands on its own, but with the integration of efforts, some progress may be made toward the solution of problems and promotion of well-being on many levels.

The description of the scope of the third wave in terms of also “beyond the individual” and focusing on “super-individual processes and phenomena” as an illustration of complexity by Lomas et al. (2021, p. 4), seems to be more in line with a wider well-being focus than what can logically be described as only part of PP. However, explaining what is meant by “going beyond the individual person as the primary focus and locus of inquiry,” the authors refer to groups, organizations, and systems that “impact upon people’s wellbeing (from politics to economics)” (Lomas et al., 2021, p. 4). The focus is thus only on human well-being and how context influences them. It does not include the behavior of humans respecting the well-being of other non-human systems for the sake of these themselves. No explicit distinction is made between a focus on the individual’s psychological well-being (as part of PP) and a focus on the well-being of broader social and contextual systems themselves as would be needed in the context of complex human-social-ecological challenges. The latter also applies to some extent to the arguments brought forward by Wissing et al. (2018, 2021). However, these authors do indicate interconnectedness as a core focus, including the ecological systems. In this manuscript, I contend that whether the focus is on how systems influence the individual or humans as a collective, or on the well-being of wider systems themselves, or the interaction among systems, the notion of interconnectedness (and specifically the quality of the interconnections) is of core importance, and will, in particular, be the case in the new post-disciplinary trajectory of well-being studies. Evolutionary and bio-cultural studies have already shown the centrality of interconnectedness for the well-being of humans and nature (Massimini and Delle Fave, 2000; Delle Fave, 2018).

Wissing et al. (2021) suggested that the transdisciplinary outgrowth characteristic of the so-called third wave with its recognition of diverse worldviews, methodologies, and focus on interconnectedness, can be compared to a butterfly leaving a cocoon - the cocoon of positive psychology being bounded by its disciplinary name. In this manuscript, it is postulated that this “butterfly” indeed signaled the development of a new domain of scientific endeavor, but that the identity (and name) of this domain needs to be further delineated and described. The butterfly metaphor can also be replaced with the idea of the new well-being domain branching out of PP, taking a new direction, while PP will continue its growth and expansion as a (sub)discipline. Such “branching out” is in line with the conceptualization by Van den Besselaar (2012/2018) of a new interdisciplinary scientific field being born on the boundaries of a discipline as one of the forms of knowledge development. This broader post-disciplinary trajectory of well-being studies proposed may also be seen as reflecting what Kuhn (1970, 1977) indicated as a change in direction, including new meta-assumptions, a coherence approach in methodology among multiple disciplines, and a new delineated focus, but the conceptualization by Van den Besselaar may be more fitting, if it is indeed a new discipline in development.

A scientific endeavor such as the hereby suggested post-disciplinary well-being studies domain that cuts across the conventional disciplinary boundaries can be seen as “convergent science” as conceptualized by Antó et al. (2021, p. 7 of 11). It consists of new ways of thinking about the process of research and the kind of strategies that are necessary for knowledge production, verification and application. Antó et al. (2021) contend that traditional universities will have to adapt in research, education, preparation for practice, and dissemination of information to include such convergent sciences. They indicate that such an approach is in particular suitable for complex issues and challenges involving more than the individual or human system (e.g., think COVID-pandemic: individuals and groups are involved from the cellular level to psychological, social, economic, political, and other human systems levels; zoological facets play a role – where did the virus originate, when can I walk the dog outside during lockdown; and ecological facets – the biosphere improve while humans were confined to their homes).

I thus conclude that there is a butterfly in the garden of well-being studies, but what does this garden look like, and can this butterfly be distinguished from others in the field? More butterfly spotters are invited to explore this question more in-depth than what is possible in the next sections as only a part of this manuscript.

### Two Trajectories – Discerning the Butterfly in the Garden of Well-Being Studies

In this manuscript, I argue thus that there will be (as far as this manuscript is concerned) two main roads ahead in well-being studies: The state of the art of PP as in the first and second waves (continuing into the present and future), and a post-disciplinary trajectory of well-being studies, initially indicated as the third wave of PP. Whereas the identity of mainstream PP is relatively well established (cf. Pawelski, 2016a,b), the identity of the post-disciplinary trajectory is still unfolding, and it is a question of how it differs from or dovetail with other streams of well-being studies with a broader focus than PP. There are several possible directions for further development of the identified post-disciplinary trajectory and opportunities to learn from other transdisciplinary endeavors and approaches. One possible direction forward is to focus from an inter- or multidisciplinary perspective on the psychological well-being of humans in context and situatedness, with the aim to understand and promote individual or group well-being as influenced by social contextual factors (e.g., Kern et al., 2020). In such a case one discipline (positive) psychology plays a dominant role. However, if the focus is not only on individual mental health and well-being, other disciplines may be more prominent or equally important. Another direction may be focusing not only on human well-being as influenced by contexts, but also focusing on the health and well-being of all non-humans, contexts, and systems themselves as evolving and changing over time and how they all hang together in sustaining life on earth. Here, the focus will include how human behavior influences the relationship with others and with animals and the ecological context to ensure that there will be a sustainable future for all and the next generations. Demarcation of focus is however necessary to have a unique character distinguishable from already existing perspectives.

#### Holistic Perspectives

There already exist various holistic inter-/multidisciplinary approaches to health and well-being, such as the One Health approach, the Planetary Health perspective, and the EcoHealth view or Global Health perspective, and more (Lerner and Berg, 2017; Rabinowitz et al., 2018; Lueddeke, 2019; Antó et al., 2021), which are all explicitly focused on systems more comprehensive than the individual person. There is as yet, no agreement about the similarities and differences among these: sometimes conceptualized as similar, but also as overlapping in ways, or as uniquely different. To a great extent, these perspectives were a follow-up on the report by the Rockefeller Foundation-Lancet Commission on “Safeguarding Human Health in the Anthropocene Epoch” (cf. Whitmee et al., 2015). This report indicated the extent to which human activities contributed to the degradation of the earth’s ecosystems and thereby threatening life on earth. The above-mentioned holistic approaches are also linked to the United Nations Sustainable Development Goals for 2030 (UN Resolution, 2015). It is not yet sure whether the new domain of well-being studies will dovetail with one of these holistic perspectives, or develop a unique focus, and whether different streams of wider well-being studies will co-develop or merge. The described third wave of PP, which is now proposed to be a new domain of well-being studies, has many characteristics similar to the above more holistic well-being perspectives, for example, a strong inter-/multidisciplinary approach and focus on broader systems than the individual person or only human systems.

To envision the character of, and further development of the post-disciplinary trajectory as identified in this study (what kind of butterfly?), the holistic health and well-being models need to be scrutinized in a bit more detail to establish to what degree the proposed “butterfly”-trajectory will fit in with them or alternatively, manifest a unique focus. In their analysis of the literature, Lerner and Berg (2017) found that the perspectives of One Health, Planetary Health and EcoHealth are sometimes used as synonyms with the same denotations, but also sometimes viewed as overlapping in some respects, and even also sometimes as sharply different from each other. The differences highlighted are as follows: The One Health movement accentuates the interrelatedness of human health, animal health, and ecosystems health, but focuses often more only on human and animal health (ignoring the health of the environment). EcoHealth strongly focuses on biodiversity, including all living creatures, and recognizes indigenous knowledge systems with a view to translate knowledge into action, but humans are less on the radar. Planetary Health has a primary “anthropocentric” view which values ecosystems mainly in terms of their impact on human health/well-being and the sustainability of life on earth. They concluded that the approaches have many similarities, and that the differences among these perspectives are reflected in the role attached to the different contributing disciplines (equal status, or one dominating in focus), and the core values expressed by the different approaches (e.g., health, biodiversity, the importance attached to humans, animals, and/or ecosystems). An overarching characteristic of all three approaches is that it is multi- or interdisciplinary in nature, based on the understanding that the problems to be solved cannot be handled by one discipline. They, however also caution that boundaries must be drawn for not taking a too wide perspective that can be seen as a “theory of everything” (Lerner and Berg, 2017, p. 6).

Rabinowitz et al. (2018) proposed a planetary perspective on health that incorporates all three of the above foci. They conceptualize three hierarchical organized pillars of humans, animals, and ecosystems, each consisting of layered systems from the minute to the most encompassing (for example, the human pillar systems are organized from the molecular level to global societies; the animal pillar from molecular to global fauna, and the ecological system from chemical/physical to the biosphere). In terms of this framework, the third wave of PP/emerging well-being studies mainly focused on the human pillar; sometimes only from the person-level to the global society (e.g., Lomas et al., 2021), but others also from the biological level to the cultural level (e.g., Massimini and Delle Fave, 2000; Delle Fave and Massimini, 2015; Delle Fave, 2018; Mead et al., 2021b). In the model proposed by Rabinowitz et al. (2018) all the system layers in a pillar influence each other hierarchically, but they are also influenced by the layers of systems in the other pillars. This framework can be applied to identify and manage health threats, but also facilitate models for well-being, healthy coexistence, and sustainability of all the implied interconnected systems. Rabinowitz et al. (2018) illustrated their conceptualization with a description of how it applies to a farming community, including humans, animals, and the local environment, and how this approach can eventually contribute to well-being of all and decrease the carbon footprint. An associated perspective is described by Lueddeke’s (2019) view of “one planet, one health, and one future.”

The focus on well-being is explicit in the perspective by Antó et al. (2021), describing how their “Planetary Well-being Initiative” is pursued in higher education. They define planetary wellbeing as “the highest attainable standard of wellbeing for human and non-human beings and their social and natural systems” and contend that “we can hope to flourish in harmony with other human and non-human beings, only through judicious attention to the political, legal, economic, cultural, and social institutions that shape the Earth’s natural systems” (p. 2 of 11). They pointed out that we need new concepts, theories, and empirical investigations to facilitate well-being for humans, animals as well as for social and natural systems. Enabling this process needs collaboration among disciplines and recognizing the complexity of the issue. In their Planetary Well-being Initiative, Antó et al. (2021) pointed out the importance of education, and illustrated the academic character of this endeavor in terms of courses presented at tertiary institutions, theme-specific conferences, publications, and more. A scholarly knowledge-to-praxis approach is noted in attention to research, teaching, and practice.

On a substantive level of focus, it is a question to what extent this new domain of well-being studies can also include attention to the consequences of major challenges as posed by displacement of peoples, climate change, zoonotic disease outbreaks, severe weather implications, and more, and how they can find balances for a greater good. This new post-disciplinary domain of well-being studies will be more limited than the holistic models. However, can individuals in this “Anthropocene” era (referring to human behavior driving the degradation of the earth’s systems) be seen as “well” if they do not also take the well-being of all non-human life, ecosystems, and biosphere in itself into account to ensure life on earth for next generations? This is not only about well-being, but also a matter of ethics. I thus foresee that the indicated post-disciplinary well-being studies domain will resonate with facets from the above holistic models, but that it will be more specific in focus and considerate of ethics-related and moral behavioral aspects.

#### More Specific Perspectives

There are also some more specific interdisciplinary approaches developed in the context of humanities and social sciences that may influence or resonate with thinking in the newly emerging domain of research on well-being. Some examples are the following: Firstly, the tripartite model by Layder (2021). He conceptualized (interdisciplinary) research as consisting of three interlocking facets: research needs a general framework reflecting the assumed global nature of social reality; a local frame or image of social reality as reflected in empirically data gathered; and a blending and processing of global and local frames via theory and methods to generate integrated explanatory accounts. This is similar to what I elsewhere in this text refer to as the “interwovenness of all the levels of the scientific text.” Other models are the interdisciplinary “conceptual engineering” model of Prinzing (2021), suggesting a constant iterative process involving normative theorizing, empirical investigation, and conceptual revision from a multidisciplinary perspective, and the GENIAL framework by Mead et al. (2021b). In the latter approach, a transdisciplinary biopsychosocial perspective is linked to ecological systems theory. These authors contend that well-being should be conceptualized broader than is the case in PP. They proposed that well-being is multifaceted, with interactions within and across multiple domains and levels. Mead and colleagues include healthy vagal functioning as part of individual well-being. They contend that connections to the self, the community, and the natural environment should be taken into account, as well as how these are influenced by external factors such as the socio-ecological context.

The recently “Emerging Science of Virtue” by Fowers and colleagues (Cokelet and Fowers, 2019; Fowers et al., 2020, 2021) is a very relevant approach to take into account in further building out of the suggested post-disciplinary well-being domain of study with its focus on values and virtuous behavior in wider contexts, and how these can foster human flourishing. These authors developed the STRIVE-4 model on virtues in intense cooperation between psychologists and philosophers. Virtues are viewed as the actualization of important values reflected in measurable traits expressed in behaviors that are fitting roles, situations, and the interaction between these. Virtues as actualized values help to attain valued ends and facilitate eudaimonic human flourishing. The model focuses in particular on habitual moral virtues such as generosity, loyalty, justice, fairness, honesty, kindness, and courage (epistemic virtues and performance virtues on the side). Each virtue is seen as consisting of virtue-related knowledge/cognition, concordant emotion/motivation, expressive of a more or less stable disposition, and reflected in behavior. They refer to “practical wisdom” (phronesis) as “the capacity to recognize the appropriate moral behavior or virtue for a given situation” (Fowers et al., 2020, p. 4). In terms of the suggested post-disciplinary domain of well-being studies, virtues will need to be also expressed in how people relate to the environment and behave in challenging circumstances. Another perspective linked to some of the above ideas is the “Science of Effective Well-Doing” described by Lieder et al. (2021) from the Max Planck Institute. They argue that the intentional pursuit of values and prosocial goals (i.e., well-doing) are of core importance for the sustainable flourishing of humanity. The new post-disciplinary field of well-being studies proposed in this manuscript resonates in many respects with ideas expressed in the above models, but will be shown to have a unique focus.

#### Learning From Other Interdisciplinary Approaches

Gervais (2021) indicated that (new) disciplines (such as the hereby proposed post-disciplinary domain of well-being studies) can benefit from taking note of developments, processes, and reforms in other disciplines to create a more optimal ecology for science. In this regard, all the holistic approaches indicated above can be of value, as well as the more delineated fields such as the emerging science of virtues. The suggested post-disciplinary domain of well-being studies can also learn from, or be modeled in line with patterns of knowledge development as described in other multidisciplinary perspectives such as that of the integral and evolving knowledge perspectives foreseen and described by Gidley (2010), Fazey et al. (2020). Fazey et al. (2020) analyzed what future transforming knowledge systems might look like and accentuated that knowledge production will need to be much more collaborative, egalitarian, open, value-sensitive, respecting values, and working with systems to generate wisdom for action. They indicated that it is not enough to generate knowledge which is often only abstract, rational and fragmented. It is necessary to also include different ways of knowing (including tacit/intuitive, experiential, and indigenous types), with the integration of knowledge and practice led by wisdom based on moral and ethical judgments about the ends pursued. They contended that their approach “reflects Aristotle’s idea of phronesis, a form of practical wisdom and knowledge where action and knowledge are oriented toward concern for human flourishing and viewed as inseparable” (Fazey et al., 2020, p. 12).

There are numerous challenges and opportunities for future well-being researchers, to explore and understand the “kind of butterfly” that emerged, and to contemplate and build out the identity of PP and the post-disciplinary development of well-being studies. Together with clarifying the focus/scope of the emerging post-disciplinary trajectory of well-being studies as indicated above, it is also essential to understand and explicate how this aligns with methodologies used and worldview assumptions. The same applies to PP as a state-of-the-art trajectory. For example, whereas Lomas et al. (2021) highlighted the scope and methodological aspects of the changing nature of PP/well-being studies, and Wissing et al. (2018, 2021) also accentuated shifts with regard to worldviews, I argue in this manuscript in particular, that the interwovenness of all levels of the scientific text (cf. Madsen, 1988) needs to be taken into account. In this process, attention needs to be given to issues ranging from broad and more abstract matters such as those pertaining to the philosophy of science and the role of worldviews (e.g., what is the nature and structure of reality and scientific disciplines, and what is the optimal social ecology for progress in science), to linked issues related to disciplinary theory and methodology, and to more narrow situated and substantive empirical foci and practice, and various others. Both trajectories offer many similar, but also widely diverging challenges and opportunities for growth and reform in knowledge production and verification. Some of these will be highlighted below, keeping an eye on the interwovenness of the various components of the scientific text.

### Worldviews – Integral Part of the Scientific Text

Both in the case of positive psychology and the post-disciplinary well-being trajectories, future researchers will need to reflect on, and take cognizance of the role of metatheoretical assumptions (worldviews) on their selected empirical foci, preferred methodologies, processes of theory-development and verification, as well as how these are intermingled and can be changing over time in the process of knowledge generation (cf. Hastings et al., 2020). Worldviews can be described as all-embracing philosophical beliefs about life and what values matter the most. Such beliefs are embedded in social and cultural contexts (Onwuegbuzie and Frels, 2016; Slife et al., 2017). Worldviews include ontological, epistemological, and axiological beliefs. Ontology is concerned with beliefs about the nature of the real world and human beings in particular. Epistemology concerns beliefs about how knowledge is generated and validated. Axiology/ethics/values is concerned with what is supposed to be good and bad/desirable and undesirable. Alexandrova (2017), as well as Hill and Hall (2018), argued that philosophical assumptions influence disciplinary conceptualizations, the methods used, the preferred methods of confirmation, and the interpretations made. Mitchell and Alexandrova (2021) contended that philosophy and disciplinary studies on well-being can benefit both when pluralism in conceptualization (on the metatheoretical level) and on theoretical and empirical denotational levels are accepted. Worldviews play a role in scientific endeavors even if not consciously recognized. It is well-known that Western perspectives in psychology and positive psychology for a long time neglected the role of worldviews and assumed that others share the same views, and that findings can be generalized to all contexts and cultures (cf. Christopher and Hickinbottom, 2008; Henrich et al., 2010) which is of course not the case.

The situation is changing, but the role of worldviews in mainstream psychology is still to a great extent not being taken into account as indicated by Slife et al. (2017), or in positive psychology as indicated by Alexandrova (2017) and suggested by Prinzing (2021) in advocating for closer collaboration between philosophy and psychology. Layder (2021) pointed out the continuing neglect of ontological issues in social sciences in general, and in particular, the consequences of views about the social reality that deeply influence theory and methods. He contended that growth in science strategically requires the intertwinedness of worldviews, theory, and methodology. He argues that the situatedness of empirical data and the global properties of what social reality is seen to be, need flexibility in research design, and in the role of theory and theory-generation in social sciences. Multidimensional, variegated models of social reality are preferred to so-called flat ontologies (as in positivist, post-positivist, and post-modern approaches). However, there are various holistic interdisciplinary perspectives on well-being as indicated in the previous section. Each has its own metatheoretical assumptions, but all indicate the intertwinedness of (implicit or explicit) worldviews, theory, and methods. Future research will need to analyze the nature of implicit or explicit approaches and distinguish the manifestations and interactions thereof with theoretical and methodological processes, which is much more than only stating that worldviews need to be taken into account.

### Theory and Methods Embedded in Meta-Assumptions

There is much room for further theory development and validation in PP and even more so for post-disciplinary well-being studies. There are some prominent theories/models in PP to which many studies refer, with some of them predating the official beginning of PP, and some focusing on measurement. Some of these are for example, Ryff’s psychological well-being model Ryff (1989, 2018), the self-determination theory of Deci and Ryan (1985, 2000), Fredrickson’s broaden-and-build model Fredrickson (1998, 2001), Keyes’s model Keyes (2002, 2007) integrating hedonic and eudaimonic facets, and many more. However, there is no strong overarching theory that can explain well-being behavior or integrate the many specific facets of well-being that are now distinguished and explored under the flag of PP. On the other hand, there are many minor theories/models or only hypotheses on fragments of well-being as related to new constructs in PP. To what extent is there a cumulative growth in well-being knowledge via theory development and validation? Future researchers in PP and post-disciplinary studies can explore what the state of the art is for them as being conducted in psychology and other disciplines. For example, McPhetres et al. (2021) found in an analysis of articles in a flagship psychology journal that the word “theory” only appears in round about half of all manuscripts, and that only 15.33% of manuscripts indicated that it is about testing of theories. They concluded that the majority of studies are not theory-driven and can thus not contribute to cumulative growth in theories. However, should all research be theory-driven, or can it also be problem-driven or discovery-oriented and still be sound science? It is important to note that not all researchers agree that empirical studies should be theory driven. This is in particular the case with studies on subjective well-being and life satisfaction as espoused by Diener and colleagues (e.g., Diener, 1984; Diener et al., 2018) who maintained a bottom-up perspective that people need to speak for themselves and not measure themselves up to externally imposed standards of what a particular form of well-being is supposed to be.

Theory development and methodological issues are closely linked as also argued in the recent debates about the reproducibility and replicability of findings in psychology. There is a concern that many findings in psychology are not reproducible. Further alarming is that non-replicable findings seem to be more often cited than replicable ones (Serra-Garcia and Gneezy, 2021). In most instances, the non-replicability problem is ascribed to methodological issues and statistical shortcomings (cf. Aarts et al., 2015; Open Science Collaboration, 2015). For example, the Open Science Collaboration (2015) group replicated 100 experiments published in top psychology journals, and found that only one-third to one-half (depending on criteria used) of the findings could be replicated. The reasons for the low percentage of reproducible findings are mostly ascribed to methodological aspects, including methods of data collection, underpowered studies, HARKing (i.e., presenting results as if the *post hoc* hypotheses - made after the results were known - as if they were the original ones), selective or inappropriate statistical analyses, p-hacking (scientists select data or statistical analyses until results become significant after initial non-significance), insufficient description of conditions necessary to obtain findings, as well as other selective or biased reporting, and more (Munafò et al., 2017; Baumgaertner et al., 2018; Efendic and Van Zyl, 2019; Muthukrishna and Henrich, 2019; Oberauer and Lewandowsky, 2019; Layder, 2021). Most of the remedies advised in the past were with regard to methodological and statistical aspects as well as a move to open, transparent science and preregistration of hypotheses and designs that may solve some of the statistical problems. Although there is strong advocacy for open science and preregistration to enhance the quality of research, not all agree that preregistration is a good idea (e.g., Szollosi et al., 2019).

However, methodological issues are closely linked to theoretical aspects. Several researchers question the idea that stricter methodological compliance rules, effect size calculations, exact p-values, other statistical approaches, or preregistered research on its own will contribute to the accumulation of knowledge and promote the growth of excellence in science, and that theory should be taken into account (e.g., Fiedler, 2017; De Boeck and Jeon, 2018; Oberauer and Lewandowsky, 2019; Szollosi et al., 2019; Layder, 2021; Scheel et al., 2021). The reforms in methodology and formalizing of hypothesis testing is valuable and should continue, but also led, according to Scheel et al. (2021), to the realization that hypotheses cannot be tested before there is an establishment of a proper and sound “derivation chain” (p. 746) between theory and the testing thereof. Oberauer and Lewandowsky (2019) suggested that there should be a distinction between theory-testing and discovery-oriented research. For theory-testing, there should, in particular, be a strong link between theory and the empirical tests thereof, whereas theoretical hypotheses play a lesser role in discovery-oriented research which is more about defining a search space and effects that could support the discovery. They suggested that a strong connection between theories and hypotheses can optimally be reached through the formalization of theories as computational models.

Muthukrishna and Henrich (2019) argued that a major component of the no reproducibility of findings is the absence of growth in theoretical frameworks that can generate hypotheses across domains (to be noted by the post-disciplinary well-being studies), and can integrate information from various disciplines. This is even lacking among the various sub-fields within psychology (and probably within PP itself also). Overarching theories (including formal modeling) may facilitate a deeper understanding of human behavior. Muthukrishna and Henrich (2019) illustrated their argument with an exposition of the explanatory power of their dual inheritance theory also known as a biocultural or gene-culture coevolution theory in which there is a genetic line (species inherit from their biological parents), and a cultural line (which is an inheritance from others in the particular society). Gervais (2021) applauded the suggested methodological reforms that are needed to take science forward, but also argued that good theory is necessary. In this regard, he indicated that there are strong multidisciplinary theories outside of mainstream psychology, such as cultural evolution theory, which may be valuable, and that modeling from the philosophy of science is necessary. He contended that large multidisciplinary research networks with more diversity included, will probably be faster in discovering the “truth.”

The lack of cumulative growth in knowledge (vs. accumulation of many disconnected empirical studies on popular topics as developed in WEIRD contexts), in psychology and other social disciplines (which may also include mainstream PP and some post-disciplinary well-being studies), is also linked to the lack of clarity about metatheoretical assumptions and the implications thereof for theory and method. Layder (2021) indicated that the distinction between theory and method is false – they are deeply intertwined and should be seen as such for problem-driven research to be undertaken. He argued that taking into account ontological dimensions and epistemological meta-perspectives on the social universe, can promote methodological foundations of evidence-based research. Diversity in perspectives and approaches, taking the complexity of worldviews and human behavior in context into account, is essential for growth in knowledge according to De Boeck and Jeon (2018) as well as Gervais (2021) and others. Devezer et al. (2019) contended that reproducibility is important for scientific growth, but also showed in the evaluation of a mathematical model, that scientific discoveries may not always be reproducible although converging to the truth, just as that reproducible findings do not necessarily converge to the truth. They also pointed out the importance of epistemic diversity to facilitate the discovery of scientific truths.

Future researchers have the opportunity to explore and evaluate the reproducibility of the many findings in positive psychology as was done in the case of organizational psychology as a (sub-)discipline by Efendic and Van Zyl (2019). The challenges are, however, not only to determine what methodological reforms are taking place or are needed, but also to develop more overarching theories, and to unravel the complex interactions and contributions of theory, methods, and worldviews in the growth of knowledge in PP as well as in post-disciplinary well-being studies. Both trajectories can benefit significantly from developments in related sciences.

### Empirical Contexts, Measures and Foci Informed by Theory and Worldviews

#### Context

Empirical data are of course, linked to theories and methods as influenced by metatheoretical assumptions and decisions – although often implicitly in existing studies. For future research, it is important for researchers in both mainstream positive psychology and post-disciplinary well-being studies, to reflect on the implications of the fact that collection of empirical data also have a very specific contextual situatedness (natural-ecological place and time historical life phase) with linked implicit social and cultural assumptions as indicated by Layder (2021). The situatedness of many well-being studies in Western contexts was often not recognized in the past, and generalizations were made as if the findings and interpretations are globally applicable (cf. first wave of PP). This had been pointed out clearly by Henrich et al. (2010) and others. The situation is changing with empirical studies being published from various continents and cultures, but still neglecting explicitly *multicultural* contexts as such, while multiculturalism and multilingualism can be a benefit to society (Wissing, 2021). There is a need for more empirical studies on well-being facets within multicultural contexts *per se*.

In mainstream PP, and in particular in the post-disciplinary well-being studies, the notion of *context* is increasingly being conceptualized more widely – as was already noted above in the description of the so-called third wave of PP. Contextual situatedness will in the future need to be more explicit concerning specific physical environments, economic contexts, socio-political and socio-demographic variables, life phases and life domains, and others. The widening conceptualization of “context” thus includes physical situatedness (such as place) but also non-physical situatedness and the interplay between them. A specific context that may play an influential role in both trajectories of well-being studies is the emerging 4th Industrial Revolution with many technological developments. An example is the Shmapped application developed by McEwan et al. (2020) which can be used for data collection as well as an intervention tool. The 4th IR is, however far more than technology in its relevance to PP and well-being studies (cf. Mayer and Vanderheiden, 2020). The expeditious developments in technology facilitate new developments in well-being studies as part of mainstream PP, as well as in the case of multidisciplinary well-being studies. Non-physical situatedness such as the social level context of power relationships, justice, values, and ethics of care will increasingly play an important role when planning studies, collecting data, and interpreting of results (cf. Di Martino et al., 2017), as well as the context of spiritual beliefs (e.g., Villani et al., 2019). Although many authors suggested that individual, social, eco-system, and spiritual levels of well-being, as well as the connections among levels, need to be taken into account (e.g., Lomas et al., 2015; Galderisi et al., 2017; Harrell, 2018; Warren and Donaldson, 2018; Mead et al., 2021b), few empirical studies had been conducted in this regard. Multimodal conceptualizations of well-being and complex contextual situatedness will need multi-, inter and transdisciplinary approaches converging in joint fieldwork for data collection in times of major challenges. Therefore, assumptions about reality and applicable theories and methods will have to be sorted out and jointly developed.

An increasingly important focus in contemporary times is the simultaneous existence of the positives and negatives of life and the dynamics involved, as shown in several studies (e.g., Ivtzan et al., 2016; Delle Fave et al., 2017; Fowers et al., 2017; Geerling et al., 2020). Most studies thus far focused only on psychological aspects, with a few (such as Ryff, 2012; Delle Fave et al., 2017; Fowers et al., 2017) taking physical/biological as well as psychological aspects into consideration. Although the second wave of PP already called attention to the consideration of both the positives and negatives in the understanding of affective and other psychological individual experiences, transdisciplinary well-being studies accentuated also wider social and ecological positives and negatives that play a role in individual well-being, but also in the health and well-being of the broader systems themselves. For example, the understanding of well-being in challenging circumstances such as the COVID-19 pandemic needs a multi-, inter- or transdisciplinary approach to understand the complexity of biological, psychological, sociological, economic, eco-diversity, and spiritual dimensions, and to manage the trauma, depression and anxiety elicited, as well as the resilience and growth that can also be noted. Mead et al. (2021a) argued the need to transcend disciplinary boundaries in handling the complexities of well-being, particularly in difficult contexts. They contended that researchers need to develop transdisciplinary models of well-being taking into account the positives and negatives of life in the interconnectedness of the individual situated in their communities and ecological contexts, taking into account the role of specific contextual factors such as the role of inequalities and culture, but also individual biological and physiological facets such as the functioning of the vagal nerve. From here, theories can be developed for behavior change interventions to improve wellbeing sustainably. Hayes et al. (2020) illustrated that a new version functional analysis combined with the evidence on change processes, integrated under an evolutionary meta-model, has direct practical value for handling complexities arising from the COVID-19 challenge. Several empirical studies focused on well-being facets such as meaning, harmony, relational aspects and/or virtues during the time of COVID-19 that elicited negative experiences and circumstances (e.g., McGrath and Brown, 2020; Arslan and Yıldırım, 2021; Carreno et al., 2021; Fowers et al., 2021; Mead et al., 2021a; Wilkie et al., 2021). Taking both the positives and negatives of life into consideration also implies that both should be evaluated and targeted in evaluation, measurement, and interventions.

#### Measures

Many studies in non-western contexts still use measures developed in Western contexts. Such *measures* may be validated in the particular non-western context, but invariance across contexts is not determined, and without exploring whether the particular construct (or translated word denoting the specific phenomenon) has the same denotation in various contexts. Many measures of facets of well-being had been developed beyond that of “subjective well-being” (positive affect and satisfaction with life), which were in the past viewed as the golden index of wellness, and target now relative more eudaimonic conceptualizations vs. earlier approaches focusing on relatively more hedonic aspects. The focus is also shifting to pluralistic measures that include apart from hedonic and eudaimonic well-being facets, also social well-being, connectedness to community, culture, governance, and nature (Mead et al., 2021b) that take the multimodalness of well-being into account in multidisciplinary approaches. Many measures within Western and non-western contexts need further validation taking the variety of physical and non-physical contexts into consideration. Future research, however, also need to include multiple methods of data gathering and non-linear and richer ways to understand and evaluate the complexity of well-being in various contexts. In post-disciplinary well-being studies assessments of the positives and negatives in human experiences, the health and well-being of wider ecological contexts, and the quality of the relatedness among humans and context, may take on many new and more complex forms than only self-rating scales. For example, using latent semantic analysis based on natural language to quantify responses to open-ended questions (e.g., Kjell et al., 2019), or implementing a non-linear lens of complex adaptive systems theory or chaos theory (cf. Resnicow and Vaughan, 2006; Bussolari and Goodell, 2009) for insight into manifestations of well-being phenomena and the dynamics thereof. Non-linear modeling (such as dynamical systems modeling, agent-based modeling, computational modeling, network modeling), or time series techniques (such as recurrence quantification analysis, phase space reconstruction, fractal and multifractal analysis), to understand complex behavioral issues (cf. Richardson et al., 2017; Pincus et al., 2018) may also play a role. However, a problem with measurement indices and quantifying analyses is that the real-life experiences of individuals that are supposed to be explored, are handled as aggregated points evaluated against an external standard that actually blur the line between a substantive psychological reality and a statistically presented reality/truth (Danziger, 1990).

#### Foci

There are many well-established constructs in the focus of PP, with extensive empirical studies on the nature, measurement and dynamics thereof, as well as many “new” constructs with related empirical explorations, and probably many more to come. For some of these, mini-theories do exist, but seldom with recognition and explication of meta-assumptions. In this section, I only want to highlight a cluster of well-being-related *foci* for which some empirical findings exist, but for which much more empirical research is needed for each of them as well as the possible underlying coherence among them and the possible integrative metatheoretical assumptions thereof. These substantive foci are *harmony, meaning, relationality and virtues*. Of course, each of them also has related phenomena and conceptualizations such as “mattering” that may link to meaning and relationality, “interconnectedness” that expresses relational aspects and balance, “harmony” that is also understood in terms of balance and peace (i.e., relational qualities), as well as “ethics and morality” which are associated with virtues and relational qualities. A brief explication of these well-being related foci and the possible links among them, will now follow:

*Meaning* (of, in, to life) has a long history of conceptualizations and empirical explorations in psychology (e.g., Frankl, 1963; Baumeister, 1991; Heine et al., 2006; Steger et al., 2009; Schnell, 2009; Wong, 2012; Delle Fave et al., 2013; Lambert et al., 2013; Martela and Steger, 2016; Baumeister et al., 2018; Baumeister and Landau, 2018; Wissing et al., 2020), and has also been contemplated on from a philosophical perspective (e.g., Morioka, 2015; Metz, 2020), but multidisciplinary work in this regard lags behind. However, Antonovsky (1987) already conceptualized and explored meaning (a component of the sense of coherence) in relation to physical health, and Ryff and colleagues (Ryff and Singer, 2000; Ryff et al., 2004) linked well-being related facets such as the experience of meaning in life and relational well-being to more healthy biological processes. There is a distinction between meaning components (e.g., coherence, purpose, and significance – Martela and Steger, 2016) and sources of meaning. Many studies link the experience of meaning in life to positive relational qualities on various levels (e.g., Lambert et al., 2013; Delle Fave and Soosai-Nathan, 2014; Wissing et al., 2019). In conceptual and empirical studies, the sources of meaning can be found to be organized and classified in various ways, but all of these include relatedness and connectedness. These connections can be interpersonal, with the society at large, or with nature and also with transcendent powers (cf. Delle Fave and Soosai-Nathan, 2014). Meaning is also linked to the notion of mattering. In Prilleltensky’s (2020) conceptualization of mattering (referring to the sense of feeling valued, and adding value to others in various contexts), he stressed the significance of balance and fairness among priorities for what is important and meaningful on personal, interpersonal, and collective well-being levels. Cooperation among perspectives from philosophy, psychology and politics is needed, and more empirical studies are needed to explore the dynamics of meaning and the outcomes. Although meaning is associated with a sense of coherence (e.g., Antonovsky, 1987; Steger et al., 2009; Martela and Steger, 2016), the collective (cohering) dimension of meaning is often neglected (Baumeister and Landau, 2018), especially in empirical studies. Keyes (1998) does conceptualize coherence as an important component of social well-being. Baumeister and Landau (2018) indicated a paucity of empirical studies on the behavioral consequences of the experience of meaning.

There is an abundance of evidence that *positive relationships and interconnectedness* as phenomena of inquiry are linked to many other facets of well-being on individual and social levels (Gable and Reis, 2010; Lambert et al., 2013; Delle Fave et al., 2016; Harrell, 2018; Warren and Donaldson, 2018; White and Jha, 2018; Algoe, 2019; Marujo et al., 2019). Often conceptualizations and empirical findings highlighted links between positive relatedness and meaning in life (e.g., Lambert et al., 2013; Delle Fave and Soosai-Nathan, 2014). Taking conceptual and empirical evidence from the natural and social sciences into account, Delle Fave and Soosai-Nathan (2014) indicated from an interdisciplinary perspective the critical role of the quality of interconnectedness on proximal, distal, and symbolic levels in shaping living systems as well as communities. They concluded that interconnectedness is at the heart of what is meaningful and that the possibility should be explored to investigate meaning from a unified interdisciplinary perspective. In their meaning and relational well-being model (M&RW), Wissing et al. (2019) offered a similar notion: meaning is made on and between various levels of reality and showed how this is empirically manifested in an African cultural context. Helne and Hirvilammi (2015), Helne (2021) proposed a strong relational conceptualization of nature-inclusive well-being and advocate for restraint in the use of the earth’s resources and a less materialistic life orientation in view of the repercussions of the rampant consumerist way of life mainly by those in well-resourced contexts. Haybron (2011) stressed the importance of the relationship between humans and nature for well-being of the former and argued that the beauty of nature has a harmonizing influence on humans, facilitating self-regulation and temperance. This idea is also contained in the arguments of Buergelt et al. (2017), indicating that humans urgently need to (re)establish a harmonious relationship with nature. Several of the studies as mentioned above explicitly frame their conceptualizations and empirical studies in the assumptions of a strong relational ontology (e.g., Helne and Hirvilammi, 2015; Marujo et al., 2019; Wissing et al., 2019; Helne, 2021).

*Virtues and moral behavior* are also linked to what is valuable for people and how these aspects play out in interpersonal relationships, as well as in wider contexts of interconnectedness. Positive relational qualities are not only linked to the experience of meaning in life, but also to harmony on individual, social and spiritual levels (Nwoye, 2018; Ohajunwa and Mji, 2018; Wang et al., 2018; Li and Düring, 2020; Wissing et al., 2020). Theoretical and empirical studies mostly explored interpersonal harmony together with intrapersonal and contextual harmony – probably because these studies are mostly linked to integrative philosophical perspectives such as Taoism, Buddhism, Confucianism, or African ontological perspectives linking people, nature and spiritual forces (e.g., Igbokwe and Ndom, 2008; Sundararajan, 2008, 2013; Nyamnjoh, 2015; Huang, 2016; Nwoye, 2018; Wang et al., 2018). From the above, it is easy to comprehend that relational qualities and harmony are also linked with *virtues*, values, peace and moral behavior, often as part of interdisciplinary studies (e.g., Fowers and Anderson, 2018; White, 2018; Fowers et al., 2020; McGrath and Brown, 2020; Fowers, 2021; McGrath, 2021; Delle Fave et al., in press). In their interdisciplinary work (psychology and philosophy), Cokelet and Fowers (2019) take a psychological realism stand and see virtues, with practical wisdom as the core feature, as measurable and that the particular environmental context will determine how these are behaviorally enacted. Virtues, morality, and ethical behavior are linked to what is considered good in all human life, but they are even of more critical importance in times of complex challenges such as those associated with climate change and the situatedness of humans in times of pandemics. Ethical behavior in research, practice, and life is always about relational qualities – with others, nature, and what is seen as important on a spiritual level. It needs to be further explored how ethical, moral and virtuous behavior hang together with harmony as a quality of well-being. Moral behavior in specific contexts may potentially give rise to disruption of harmony in specific situations. How will these processes play out to be handled in a balanced manner? Some clues may be found in the processes of self-regulation and temperance, as suggested by Haybron (2011), Van Tongeren et al. (2018) and reviewed by Worthington and van Zyl (2021), and the virtues perspective by Cokelet and Fowers (2019), Fowers et al. (2020).

*Harmony* as a phenomenon received much attention from philosophy particularly in East Asian (e.g., Li, 2016; Wang et al., 2018; Li and Düring, 2020) and African (e.g., Igbokwe and Ndom, 2008; Mkhize, 2008; Metz, 2016) contexts, but empirical studies lagged behind, especially in Western contexts. Only in recent times harmony and associated constructs and processes such as balance, peace, serenity, harmonization and more, came into focus in scientific psychological studies on well-being referring to qualities on intrapersonal, interpersonal, social, ecological and spiritual levels. Literature reviews (e.g., Wallace and Shapiro, 2006; Lomas, 2021; Delle Fave et al., in press), the conceptualization of models (e.g., Di Fabio and Tsuda, 2018; Gruman et al., 2018; Sirgy, 2019), and empirical studies (e.g., Chuang, 2005; Sirgy and Wu, 2009; Delle Fave et al., 2011, 2016; Lam et al., 2012; Kjell et al., 2016; Schutte et al., 2021) attest to the link between harmony and many facets of well-being, with some placing harmony and balance at the core of functioning well. For example, in a multi-country study, Delle Fave et al. (2016) found that inner-harmony and relational connectedness are core components of what laypeople see as happiness. In an overview integrating philosophical, conceptual, and empirical findings on harmony as explored in psychology, Delle Fave et al. (in press) concluded that harmony might share features with several virtues, aspects of meaning and meaning-making, and the quality of relatedness/interconnectedness. Based on a literature review of mental health, Wallace and Shapiro (2006) concluded that mental balance could be seen as the core of well-being. Similarly, Lomas (2021) concluded from a narrative literature review that balance and harmony are at the heart of well-being manifested in any or all dimensions or facets of life. Sirgy (2019) described a comprehensive hierarchical model of positive mental health guided by the idea of positive balance. He defined positive mental health as a positive balance referring to “a preponderance of a desirable state over an undesirable state specified uniquely at each level of analysis” (Sirgy, 2019, p. 2 of 10), with levels of analysis being indicated as physiological, emotional, cognitive, meta-cognitive, developmental, and social-ecological. Conceptualizations of harmony and harmonization as central to well-being and sustainability of life on earth is explicitly indicated by various researchers, for example, Di Fabio and Tsuda (2018) proposed harmony and harmonization processes at the individual, group, social, and national levels as vital for sustainable development and even suggested that this may form a new research area in psychology. Jordan and Kristjánsson (2017) proposed “harmony with nature” as a specific virtue, building on Aristotle’s virtue ethics, and seeing the world in terms of relationships, connections, and context. Harmony and harmonization as ceaselessly changing processes evolving over time are thus strongly linked to notions of meaning, relationality, and virtues (Kwan et al., 1997; Lambert et al., 2013; Jordan and Kristjánsson, 2017; Di Fabio and Tsuda, 2018; Wissing et al., 2020; Delle Fave et al., in press) and its manifestations proposed on and among multiple dimensions from intra- and interpersonal to social, ecological and spiritual levels. Various measures and evaluation strategies were developed to evaluate (facets of) harmony and balance based on different theories and metatheoretical assumptions, for example by Kwan et al. (1997), Lee et al. (2013), Bell and Mo (2014), Igbokwe et al. (2015), Kjell et al. (2016, 2019), but a comprehensive index of situation and context-relevant harmony and harmonization processes on and among all levels is still lacking.

Based on the above analysis of the constructs referring to meaning, relationality, virtues and harmony, and the highlighted overlaps among them it is suggested that they may hang together in a cohering nomological network, still to be explored on an empirical level. Apart from the overlapping denotations of these constructs, the possible wider cohering function of harmonization stands out because of the explicit references made in perspectives on harmony to the quality of relatedness also with non-humans and with nature so relevant in these challenging times of climate change and pandemics. Future research in disciplinary and, in particular, post-disciplinary trajectories outlined above, can explore the empirical links and dynamics among harmony, meaning, positive relatedness and virtues in various contexts. The aim will be to investigate and conceptualize some possible deeper underlying structures and processes that link these phenomena/concepts on individual person, social/collective and contextual levels.

## Cohering Harmony as Focus for Post-Disciplinary Well-Being Studies?

The content of this section should be read together with the above exposition of the so-called third wave of PP and notions about the butterfly in the garden of well-being studies referring to the characteristics of what is proposed as a new domain of well-being studies. In this section the structure and possible unifying focus for this domain will be considered. Delle Fave et al. (in press) suggested that harmony as a phenomenon may be a core dimension of human functioning, but that much more conceptualization and empirical studies are needed from interdisciplinary perspectives to develop a unified view. From the analysis in the preceding sections, it seems that the proposed new domain of post-disciplinary well-being studies can be structured around a focus on the quality of interconnectedness of human and non-human systems and the complexity of dynamics among them. Therefore, it may be that harmony (as the quality of in-between-ness) can be postulated as a core focus for such an inter- or transdisciplinary domain of well-being studies. However, from the analysis in the previous section of the constructs referring to meaning, relationality/interconnectedness, virtues and harmony and the associations and overlaps in denotations among them, it seems that there may be a broader overarching (or underlying) cohering phenomenon at play which may be more than an overlap of meanings (in linguistic sense) on the level of individual experiences, and which may open up a space for a focus in multi-, inter-, or transdisciplinary well-being studies regarding cohering processes on and among individual, social, and ecological levels. For the time-being, this hypothesized broader network phenomenon/construct is called “cohering harmony” (in which ethical behavior is required for a greater good). It is proposed as a possible unique focus for well-being studies cutting across disciplines. Cohering harmony is more complex than what was traditionally seen as harmony defined in terms of intra- or interpersonal well-being experiences for the good of the individual or human systems alone – the well-being of non-humans and natural systems themselves are also at stake in the presently proposed cohering process where survival of all life on earth is viewed as important. Further conceptualization and empirical studies are of course, indicated for clarification and elaboration, and for the time-being, the terms “harmony” and “cohering harmony” may be used as similar. For the present purposes cohering harmony and harmonization as well-being phenomenon and process is defined as an in-between quality and dynamic of relatedness that evolve and change over time and contexts, as meanings are made in ceaselessly changing relationships within and among people, and between people and non-humans and ecological contexts as expressed in virtuous behaviors and balancing of interests toward the good of humans, non-humans, and nature.

The *focus* of the emerging post-disciplinary domain of well-being studies (COHAR = cohering harmony), born on the boundary of PP as (sub)discipline and initially named “the third wave of PP,” is conceptualized in terms of main research questions rather than content of traditional academic subject areas as is described by Van den Besselaar (2012/2018) being the case for inter- or transdisciplinary studies. The main questions guiding research in this post-disciplinary domain of well-being studies (COHAR) may be regarding the complexities of balancing and harmonizing the interest of human and non-human systems toward healthy sustainable functioning and the greatest good for all, and how cohering processes may be optimized over time and during changing conditions toward well-being, while taking contextual, cultural, political, social, economic and individual situatedness ethically into account. This may include, for example, how cohering harmony processes will be applicable, manifest or be promoted in the context of differential effects of the climate crises and climate mitigation efforts on people in different socioeconomic contexts and situatedness (such as inequity, extreme poverty, violence, rural vs. urban living, and more – cf. Thomas et al., 2019). The details of a specific research project will be determined in conversations among researchers from different disciplines in equal status (or rotating leadership depending on the specific research) guided by research questions. Depending on the specific study, several disciplines from across the academic spectrum, may be part of such an endeavor in varying combinations. But of course, working in multi- inter- or transdisciplinary studies require mutual trust as such endeavors have their own challenges in terms of worldviews, conceptualization, terminology, methods used, and interpretations (cf. Fowers, 2021). The focus on cohering processes in and among various systems as proposed for the new domain of well-being studies, link to notions expressed by Rabinowitz et al. (2018) in which hierarchical and lateral links are considered in and among human and non-human systems, and the perspective of Mead et al. (2021b) arguing in their transdisciplinary biopsychosocial model linked to ecological systems theory, that connections to the self, community and natural environment needs to be taken into account, and that such studies are more than what can be called PP alone. Conceptualizations in this post-disciplinary domain of well-being studies also link to notions expressed in the science of virtues (Fowers et al., 2020, 2021; McGrath and Brown, 2020) and ideas of effective well-doing by Lieder et al. (2021), and dovetails with perspectives on nature-inclusive well-being for sustainability (e.g., Kjell, 2011; Helne and Hirvilammi, 2015; Horton and Horton, 2019; Cianconi et al., 2021; Helne, 2021).

Taking the above conceptualization of focus to the ground: Harmony and balance had been shown in empirical studies as important phenomena on intrapersonal, interpersonal, and social levels, but also as an essential quality of the link between humans and natural contexts, and within ecological contexts themselves that also need to be healthy and well. To reach (cohering) harmony as a desired quality of interconnectedness, virtues as expressed in behaviors, play an important role. In this perspective, well-being will mean that human beings will act virtuously and caringly, not only to themselves and each other, but also toward the earth, for example as shown in efforts to stop the degradation of the environment and biosphere, restrain in use of natural resources just for their own benefit, and to compassionately also care for animals and the environment while also enjoying fulfilling and warm interpersonal relationships and a deeply respectful and wondering attitude toward nature, letting the biodiversity flourish. This attitude toward the environment is already known as environmental ethics. Such an approach that includes moral behavior takes hand with research on character strengths as conceptualized in PP literature (cf. Peterson and Seligman, 2004; Niemiec and Pearce, 2021). In the post-disciplinary field of well-being studies as conceptualized in this manuscript, aspects such as interconnectedness/relatedness, virtues/morality/ethics, and meaning of, in, and to life, are all deeply interwoven in the concept of cohering harmony and harmonization on a broader level than only the individual person and in social systems. Cohering harmony, balance, and harmonization on intrapersonal, interpersonal, social, and ecological levels as well as on the in-between quality among all systems and levels are relevant.

With regard to *epistemological and methodological approaches*, the proposed post-disciplinary domain of well-being studies as described above, may resonate with ideas from the future transforming knowledge productions processes as highlighted by Fazey et al. (2020). They indicated that knowledge production is viewed as collaborative, open, egalitarian and led by values, taking various systems into account while generating wisdom to integrate knowledge and practice based on moral and ethical judgments about the ends pursued. Such endeavors may include academics from various disciplines, but also other stakeholders as well as laypeople. A plurality of methodologies may be used in research designs and the collection of empirical data. It will aim to develop theories for an explanation that can be verified in further research, and seek to integrate knowledge and practice with, and toward wisdom, while knowing that knowledge development is an ongoing and changing process itself.

Post-disciplinary well-being studies may be conducted from a plurality of *metatheoretical perspectives*, including a relational, interconnectedness and virtue ethics perspective. Cohering harmony as a unified focus for post-disciplinary well-being studies presupposes a strong relationality worldview assuming the interconnectedness of all systems on earth and the requirement for morally responsible behavior not only toward humans and human systems, but also to non-humans and broader ecological systems on earth for the sustainability of life and for future generations. Such a post-disciplinary approach in well-being studies may compel researchers to take a fresh look at their disciplinary assumptions, reconsider familiar concepts and methods in order to cohere and develop a deep integration in the joint research, and invest themselves fully and equally in projects from the beginning to the end.

## Conclusion

This manuscript argues that the so-called “third wave of positive psychology” was actually the beginning of a new scientific domain of well-being studies with a multi- or interdisciplinary nature, wider focus, and inclination to answer the call for action in complex situations and changing times. As such, this post-disciplinary trajectory of well-being studies is more than PP as a (sub)discipline as initially described by Wissing et al. (2018), Lomas et al. (2021). However, the jury is not out on this matter as yet. It can be argued that the goal and scope is the determining element: If multidisciplinary studies are focused only on *psychological* human experiences and behaviors as influenced by contexts and wider systems, it might be conceptualized as part of PP (but why not psychology?). If the multidisciplinary studies, with a more equal status among the contributing disciplines, focus also on the well-being of wider systems themselves and the interconnectedness of all of these, it may be a misnomer to call it PP. It is rather a matter of a new inter- or transdisciplinary domain of scientific well-being studies that dovetail with more holistic perspectives such as One Health, or Planetary well-being, but with a more limited scope and links to other social and humanity perspectives as the emerging science of virtues indicated above. The strong post-disciplinary nature of well-being studies, and consideration of the well-being of many systems to ensure sustainability, and linking values, theories, methodology and empirical studies, indicates that it is beyond PP as (sub)discipline, and that a broader perspective is opening up on well-being. It is therefore foreseen that there will be (for now) at least two trajectories of well-being studies: mainstream PP with an in-depth focus on (fragmented) well-being facets and processes, and a post-disciplinary scientific domain with a delineated wider focus and more capability to address complex challenges of health and well-being in an integrated manner. In-depth studies in mainstream PP will continue and also feed into post-disciplinary well-being studies, with the latter increasing in importance. It may also be that PP as a (sub)discipline of psychology become increasingly integrated into psychology as discipline, enriching psychology with a balanced consideration of positive and negative features of life and events. Thoughtful reflection is invited on the identity, goals and road ahead for each of these trajectories.

There are many similar but also diverging challenges and opportunities for future research in both trajectories. An overriding challenge indicated for both streams of research is to take all levels of the scientific text into account, and in particular the interwovenness among them. The mainstream PP had neglected worldviews and consequences for a long time (but it is changing now – cf. Clifton et al., 2019). In contrast, the post-disciplinary trajectory was more explicitly mindful of ontological and epistemological assumptions and the role of values from its emergence. Both trajectories need more comprehensive and explanatory theories linked with worldviews and situatedness of phenomena. There are many more minor theories linked to specific constructs in mainstream PP, but no overarching or strongly integrative theories. More integrative biocultural and evolutionary theories are often proposed in the post-disciplinary stream, and an Aristotelian virtue ethics meta-perspective is taken. Both trajectories need to take the situatedness of empirical information into account and renew methodologies for more trustworthy findings. A specific substantive focus highlighted for further research is the exploration of the underlying coherence and dynamics of harmony, meaning, relatedness, and virtues on individual, collective and wider system levels – the latter is a new perspective to be explored further.

In this manuscript, a cohering core for the post-disciplinary well-being domain is highlighted, referring to its possible structure across disciplines, focus, methodologies and metatheoretical perspectives. It is suggested that “cohering harmony” and harmonizing or balancing processes can be (or already is) the central focus of this proposed post-disciplinary domain of well-being studies. This post-disciplinary well-being research domain cuts across the conventional disciplinary boundaries and can be seen as a “convergent science” as conceptualized by Antó et al. (2021, p. 7 of 11). Further conceptualization and empirical studies including multiple disciplines can articulate the processes and actions to facilitate such cohering harmony, while taking into account metatheoretical assumptions, theoretical and methodological aspects, and the specific local empirical situatedness of observation and data collection.

The identified post-disciplinary trajectory can serve as an anchor for studies on virtuous behavior and orientations focusing on cohering harmony and the harmonization of interconnectedness conduits within and between individuals and other people, non-humans and ecological systems, and can provide hypotheses inviting further research. Just as the emerging science of virtues cannot be subsumed by PP despite some links to it (Fowers et al., 2020), the post-disciplinary well-being trajectory (delineated in this manuscript) focusing on the quality of interconnectedness, cohering harmony and balance in and among various life systems cannot be subsumed in PP, despite its links to PP in its emergence. Developments within mainstream PP and other disciplines will, however, continue and feed into this new domain of well-being studies. The intentional pursuit of harmony and balance in all relational components/conduits of interconnectedness as the goal and virtuous implementation of relevant values may serve humans and well-being on the earth good in the long run. Future researchers are invited to take this perspective further. How the state of the art PP trajectory and the post-disciplinary trajectory of well-being studies will develop, time will tell. This development will be further steered by the assumptions, foci and efforts of the next generation of well-being researchers and a transformation in thinking about well-being, and how to understand and promote it in context and on a broader ecological systems level, especially in times of enormous challenges and changes taking the interests of all stakeholders into account.

## Data Availability Statement

The original contributions presented in the study are included in the article/supplementary material, further inquiries can be directed to the corresponding author.

## Author Contributions

The author confirms being the sole contributor of this work and has approved it for publication.

## Conflict of Interest

The author declares that the research was conducted in the absence of any commercial or financial relationships that could be construed as a potential conflict of interest.

## Publisher’s Note

All claims expressed in this article are solely those of the authors and do not necessarily represent those of their affiliated organizations, or those of the publisher, the editors and the reviewers. Any product that may be evaluated in this article, or claim that may be made by its manufacturer, is not guaranteed or endorsed by the publisher.
